# Direct hypothalamic and indirect trans-pallidal, trans-thalamic, or trans-septal control of accumbens signaling and their roles in food intake

**DOI:** 10.3389/fnsys.2015.00008

**Published:** 2015-02-13

**Authors:** Kevin R. Urstadt, B. Glenn Stanley

**Affiliations:** ^1^Department of Psychology, University of MichiganAnn Arbor, MI, USA; ^2^Departments of Psychology and Cell Biology and Neuroscience, University of California – RiversideRiverside, CA, USA

**Keywords:** accumbens, septum, paraventricular thalamus, ventral pallidum, lateral hypothalamus, eating

## Abstract

Due in part to the increasing incidence of obesity in developed nations, recent research aims to elucidate neural circuits that motivate humans to overeat. Earlier research has described how the nucleus accumbens shell (AcbSh) motivates organisms to feed by activating neuronal populations in the lateral hypothalamus (LH). However, more recent research suggests that the LH may in turn communicate with the AcbSh, both directly and indirectly, to re-tune the motivation to consume foods with homeostatic and food-related sensory signals. Here, we discuss the functional and anatomical evidence for an LH to AcbSh connection and its role in eating behaviors. The LH appears to modulate Acb activity directly, using neurotransmitters such as hypocretin/orexin or melanin concentrating hormone (MCH). The LH also indirectly regulates AcbSh activity through certain subcortical “relay” regions, such as the lateral septum (LS), ventral pallidum (VP), and paraventricular thalamus, using a variety of neurotransmitters. This review aims to summarize studies on these topics and outline a model by which LH circuits processing energy balance can modulate AcbSh neural activity to regulate feeding behavior.

## Introduction

### The lateral hypothalamus

The rise in obesity rates in the U.S. (Flegal et al., 2002) and other developed nations has made an urgent case for research to unravel the brain’s regulation of hunger and motivation to conume foods. Through this research, various brain areas have been implicated in food intake control. One area that has received much focus in this field is the lateral hypothalamus (LH). Once dubbed the “hunger center” of the brain, the LH has since been characterized as a signal-integrating hub for diverse neural inputs that regulate its output—the initiation of food procurement and consumption (Wise, 2013).

Many functional studies of the LH have revealed its impact on feeding. For instance, electrical stimulation of the LH elicits intake (Wyrwicka and Dobrzecka, 1960), while electrolytic lesions of the LH induce aphagia (Winn et al., 1984). Electrophysiological monitoring of LH neurons during certain food-related activities provides additional insight for the LH’s role in integrating food-related sensory information. LH neurons change firing rates to visual food cues (Mora et al., 1979) and to decreases in blood glucose (Oomura et al., 1974), while eating reverses such changes in firing rates (Mora et al., 1979). This collection of evidence highlights the LH’s role in feeding behaviors.

Various behavioral studies have delineated subdivisions of the LH into distinct feeding-regulating ensembles. These subdivisions include the perifornical area (pfLH; Leibowitz and Rossakis, 1978), the area lateral to the fornix and adjacent to the internal capsule/cerebral peduncle (lLH; Stanley et al., 1993a), and the area ventrolateral to the fornix and adjacent to the optic tract (vlLH) (Baldo et al., 2004). The pharmacological underpinnings of food intake control via the pfLH and the lLH are well-established. The lLH is particularly receptive to primary amino acid neurotransmitters; injection of glutamate, glutamate agonists, or GABA_A_ receptor antagonists specifically induces feeding (Stanley et al., 1993a; Duva et al., 2002; Turenius et al., 2009a,b; Charles et al., 2014). Also, microdialysis within the lLH reveals increased synaptic glutamate and decreased synaptic GABA prior to onset of a meal, while the reverse occurs prior to cessation of a meal (Rada et al., 1997). Thus, a balancing of synaptic glutamate and GABA input to lLH neurons is utilized to direct feeding in a minute-by-minute fashion (Stanley et al., 2011). The pfLH is a locus through which NPY, norepinephrine, and dopamine regulate food intake (Leibowitz and Rossakis, 1978, 1979; Stanley et al., 1993b) in addition to other neurotransmitters (for review, see Meister, 2007). The behavioral effects of vlLH manipulations are not well characterized. Though functional studies support these three general LH subdivisions, anatomical evidence further subdivides these areas into numerous smaller regions that may also be functionally distinct (Swanson, 2004; Swanson et al., 2005).

The LH also regulates effort to procure food rewards. Two neuron groups in particular, the orexin and the melanin concentrating hormone (MCH) neurons, are distributed throughout the LH (Hahn, 2010) and mediate consumption and/or food-directed effort-based actions. ICV injection of orexin or MCH induces food intake (Rossi et al., 1999; Zhu et al., 2002). Orexin neurons are activated by contexts associated with food rewards. Such contexts feature either sweet foods for satiated rats or regular chow for food-deprived rats (Harris et al., 2005; Choi et al., 2010). Further, ICV injection of orexin increases progressive ratio responding breakpoints for palatable food rewards, whereas injection of its antagonist decreases this breakpoint (Choi et al., 2010). In a related manner, MCH directs reward-motivated behaviors. MCH knockout mice and wild type mice given an MCH antagonist display impairments in selectively responding to reward-associated cues, showing how MCH inhibits generalization of responses to non-rewarding stimuli (Sherwood et al., 2012). It should be noted that unlike orexin, MCH originates from extra-hypothalamic areas such as the basal forebrain and pons (Bittencourt et al., 1992). Thus, it is unclear how these other MCH groups contribute to behaviors manifested by ICV MCH injections. Nonetheless, orexin and MCH can regulate both feeding and food reward procurement in complimentary ways.

LH innervation of mesolimbic circuitry serves to integrate homeostatic information with motivation to procure foods and the valuation of food tastes. Indeed, food deprivation increases “liking” responses while caloric satiety decreases them (Berridge, 1991). Also, ablation or inhibition of the anterior LH will suppress “liking” responses to sucrose and reduce food intake (Ho and Berridge, 2014). LH orexin neurons are one likely candidate population that may at least modulate some of these effects. Orexin neurons are activated by deficits in available glucose (Nishimura et al., 2014) as well as by food restriction (Kurose et al., 2002), and are inhibited by direct application of glucose (Burdakov and González, 2009) and leptin (Yamanaka et al., 2003). Aside from direct effects on orexin neurons by circulating factors (Hâkansson et al., 1998; Burdakov and González, 2009; Qi et al., 2010), some effects may be mediated indirectly via the arcuate nucleus and its peptides (Zheng et al., 2002), via long-form leptin receptor-possessing (LepRb) neuron innervation of orexin neurons (Louis et al., 2010), and/or via direct action of leptin on orexin neurons.

### The nucleus accumbens shell

Another brain region, the nucleus accumbens, is most typically associated with hedonia and motivation (Broekkamp et al., 1975; Kelley et al., 2002) yet also regulates the conversion of motivation into goal-directed actions (Mogenson et al., 1980). Rats self-stimulate when an electrode is implanted into the accumbens (Schaefer and Michael, 1992). Accumbens neurons can fire to the receipt of a reward or in reward-associated contexts (Apicella et al., 1991a,b), and some accumbens neurons produce action potentials in anticipation of a reward (Schultz et al., 1992). Activation of accumbens neurons also occurs in animals re-exposed to drugs of abuse after withdrawal and abstinence (Todtenkopf et al., 2002). Further, the accumbens innervates components of the basal ganglia (Mogenson et al., 1983), and alterations in accumbens activity change exploratory behaviors (Mogenson and Nielsen, 1984), implicating this area in initiation of goal-directed actions. These are a select few of many studies implicating this region in reward perception and motivated behaviors.

However, the accumbens also regulates feeding, particularly through the accumbens shell (AcbSh) subregion. Lesioning the AcbSh increases food intake, while AcbSh stimulation decreases it (Ramaswamy et al., 1998; van der Plasse et al., 2012). Activation of AcbSh GABA, mu opioid, or δ opioid receptors, or blockade of AcbSh AMPA receptors, induces feeding specifically (Maldonado-Irizarry et al., 1995; Stratford and Kelley, 1997; Stratford et al., 1998; Castro and Berridge, 2014a). Also, AcbSh neurons pause firing prior to initiation of drinking sucrose solution (Krause et al., 2010), synaptic glutamate levels within the AcbSh decrease during onset of eating (Rada et al., 1997), and AcbSh neurons are activated by contexts associated with palatable meal availability (Park and Carr, 1998). Further, rats recently fed to satiety show reduced expression of preproenkephalin within the AcbSh, signifying a decrease in intra-AcbSh synthesis of the mu opioid receptor (MOR) ligand enkephalin (Will et al., 2007). These data indicate a role for the AcbSh in controlling feeding behaviors.

Glutamate, GABA, and opioid neurotransmission in the AcbSh does not solely affect food intake, but instead acts on the motivation to procure food, the motivation to eat food, and/or hedonic valuation of foods. AcbSh GABA_A_ receptor activation or MOR activation increases operant responding for food reward (Wirtshafter and Stratford, 2010; Stratford and Wirtshafter, 2012a), suggesting that these receptors govern the motivation to procure foods. Although AcbSh AMPA receptor blockade does not affect progressive ratio responding breakpoint for food reward, AMPA receptor activation decreases this breakpoint (Mena et al., 2013), suggesting that AcbSh glutamate negatively modulates food-directed efforts. Altering activity of each of these receptors within the AcbSh influences food intake in satiated rats (Maldonado-Irizarry et al., 1995; Stratford and Kelley, 1997; Zhang et al., 1998), demonstrating the roles of these receptors in the motivation to consume foods. However, these receptors have dissociable roles in modulating the rewarding value of foods. Specifically, GABA_A_ or opioid receptor activation increases hedonic value of foods (Peciña and Berridge, 2005; Castro and Berridge, 2014a), whereas AMPA receptor blockade does not (Faure et al., 2010).

Interestingly, these motivational and hedonic effects differ between anterior and posterior AcbSh subregions (aAcbSh and pAcbSh). Activation of GABA_A_ receptors within the aAcbSh will increase food consumption and “liking” reactions to sweet tastes. In contrast, GABA_A_ receptor activation within the pAcbSh will reduce food intake, suppress “liking” reactions to sweet tastes, and increase aversive responses to bitter tastes (Reynolds and Berridge, 2001, 2002; Faure et al., 2010). Opioid receptor activation within the aAcbSh or the pAcbSh alters hedonic responses to sweet taste similarly to GABA_A_ receptor activation, though food intake effects differ between opioid receptor subtypes (Castro and Berridge, 2014a).

In a complimentary manner, AMPA receptor blockade in the aAcbSh increases food intake while in the pAcbSh such blockade decreases intake, though no effects on “liking” or “disliking” are observed from this manipulation (Faure et al., 2010). However, the testing environment can re-adjust this rostrocaudal difference in AcbSh behavioral control. In a normal testing environment, aAcbSh and pAcbSh DNQX administrations produce appetitive and avoidance behaviors, respectively. Testing in the quiet, dark, familiar home cage causes DNQX injections to all but the caudal 10% of the AcbSh to induce appetitive behaviors, whereas testing in a noisy, bright, unfamiliar environment causes DNQX injections to all but the rostral 10% of the AcbSh to induce aversive behaviors (Reynolds and Berridge, 2008). Thus, environmental stressors can “re-tune” the AcbSh AMPA receptor-mediated neuroanatomical sites governing appetitive and aversive behaviors.

Another intriguing finding is that blockade of metabotropic glutamate receptors 2 and 3 (mGluR2/3) in most of the AcbSh suppresses food intake and hedonic reactions to sucrose while increasing defensive treading and aversive reactions to sucrose (Richard and Berridge, 2011). As AcbSh mGluR2/3 activation decreases extracellular glutamate and mGluR2/3 blockade increases it (Xi et al., 2002), mGluR2/3 function as a pre-synaptic “brake” on glutamate release into the AcbSh. Considering these data, increased glutamate efflux to both the aAcbSh and pAcbSh can blunt motivation to consume food and can reverse tastes from palatable to aversive. Indeed, activation of AcbSh AMPA receptors decreases sucrose intake (Stratford et al., 1998). In contrast, decreased glutamate input to the AcbSh (mimicked by AMPA receptor blockade) only acts on motivation to eat, and does so in a neuroanatomically “fluid” manner dependent on the presence of environmental stressors.

In summary, GABA and opioid neurotransmissions to the AcbSh regulate food directed effort, the motivation to eat, and the hedonic and aversive properties of food tastes. These neurotransmitters perform these behavioral functions oppositely within the aAcbSh vs. the pAcbSh. Hedonic and motivational effects of glutamate neurotransmission to the AcbSh are more nuanced. Excess glutamate neurotransmission to most of the AcbSh hinders motivation to procure and to consume food and reverses hedonic perception of sweet tastes to aversive. In contrast, decreased AcbSh glutamate input acts to alter “wanting” without altering “liking”. Stressors substantially alter which AcbSh subregions, in response to decreased glutamate, produce appetitive vs. avoidance behaviors. These changes in the motivation to eat and the rewarding value of foods are transmitted to downstream behavioral effector regions, including the LH.

## Descending AcbSh to LH circuits

Anatomical evidence reveals a direct projection from the AcbSh to the LH (Usuda et al., 1998; Duva et al., 2005). In particular, the dorsomedial aAcbSh “hedonic hotspot” projects strongly to the anterior vlLH and the pfLH, likely innervating orexin neurons, whereas the ventromedial aAcbSh projects predominantly to the lLH (Yoshida et al., 2006; Thompson and Swanson, 2010; Zahm et al., 2013). Thus, the aAcbSh “hotspot” is poised to regulate hedonic value of tastes through pfLH orexin neurons, while the ventromedial aAcbSh likely drives food intake through the primary amino acid neurotransmitter-sensitive lLH.

**Figure 1:**
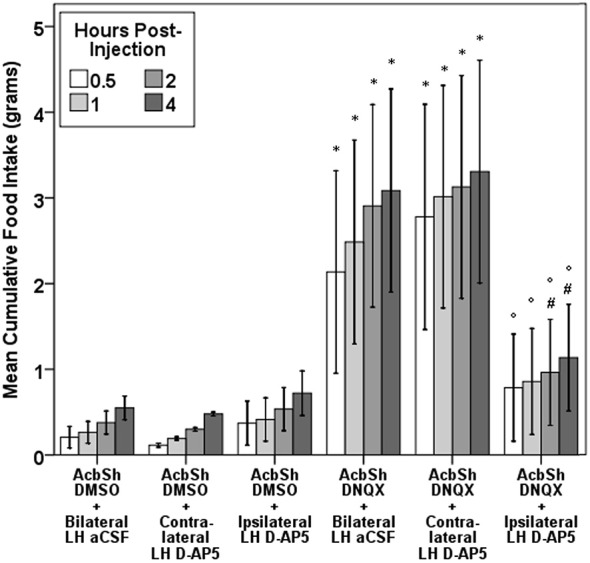
**Evidence for a feeding-specific connection between the AcbSh and the LH**. Unilateral aAcbSh injection of the AMPA receptor antagonist DNQX (0.75 µg in 0.3 µL of a mixed DMSO-artificial CSF vehicle) significantly increases feeding. This feeding is suppressed by concurrent administration of D-AP5 (2 µg in 0.3 µL of artificial CSF) into the LH of the ipsilateral, but not contralateral, brain hemisphere. This figure was duplicated from a prior study (Urstadt et al., 2013a).

Multiple functional studies support this AcbSh to LH circuit model. AcbSh GABA- or glutamate-mediated feeding is suppressed or halted by pharmacological inhibition of the LH (Maldonado-Irizarry et al., 1995; Stratford and Kelley, 1999) or by LH lesions (Stratford and Wirtshafter, 2012b). Further, unilateral LH lesions or unilateral lLH inhibition reduces AcbSh feeding in a behaviorally-specific manner (Stratford and Wirtshafter, 2012b; Urstadt et al., 2013a,b). Indeed, feeding induced by unilateral AcbSh AMPA receptor blockade with DNQX is suppressed by NMDA receptor blockade of the ipsilateral but not contralateral LH (Figure 1), demonstrating that unilateral LH inhibition specifically suppresses AcbSh mediated feeding. As the projection neurons of the AcbSh are primarily GABAergic (Oertel and Mugnaini, 1984), some suggest AcbSh inhibition disinhibits the LH, allowing for feeding to occur (Kelley et al., 2005a) and increasing motivation to procure foods (Wirtshafter and Stratford, 2010). aAcbSh inhibition with muscimol results in increased c-fos expression in the pfLH, lLH, and vlLH subregions (Stratford, 2005), and particularly in orexin neurons but not in MCH neurons (Zheng et al., 2003; Baldo et al., 2004). Based on these data, AcbSh-mediated feeding is regulated through multiple LH subregions.

Some recent data implicates the ventral pallidum (VP) as an indirect route for AcbSh to LH signaling in the control of food intake. Anterograde tracing studies have shown that the AcbSh projects to the medial VP (Usuda et al., 1998) and the medial VP in turn projects to the LH (Groenewegen et al., 1993). Functional evidence measuring food intake also supports a model of this three-part circuit. Feeding elicited by unilateral AcbSh muscimol injection is halted by excitotoxic lesion of the ipsilateral VP or the ipsilateral LH (Stratford and Wirtshafter, 2012b). Also, unilateral AcbSh muscimol induces c-fos expression in both the ipsilateral VP and the ipsilateral LH (Stratford, 2005). How this indirect AcbSh—VP—LH circuit operates in parallel with the direct AcbSh to LH circuit to control feeding remains unclear. Also, the neurotransmitters used in this three-part circuit are only partly described; AcbSh projections are GABAergic (Oertel and Mugnaini, 1984), but the VP may secrete either GABA or acetylcholine (Walaas and Fonnum, 1979) and opioid co-transmitters (Harlan et al., 1987) to the LH.

## Direct ascending pathway from LH to AcbSh

Evidence for a direct LH to AcbSh projection also exists. Early retrograde tracing studies using AcbSh injections of Fluorogold or wheat germ agglutinin revealed AcbSh-projecting neurons in the pfLH, lLH, and vlLH (Phillipson and Griffiths, 1985; Brog et al., 1993). Moderate numbers of retrogradely-labeled neurons were observed within the pfLH and lLH after injections in either the medial aAcbSh or the medial pAcbSh. In contrast, numerous vlLH neurons were labeled by pAcbSh tracer injections while few were labeled by aAcbSh injections. These anatomical findings suggest that two distinct LH populations differentially innervate the AcbSh, one arising from the pfLH and lLH, the other from the vlLH.

Due to differences in the neurotransmitters used by each subregion, the pfLH/lLH and the vlLH differently regulate AcbSh activity. The pfLH and lLH secrete a myriad of peptide neurotransmitters in addition to orexins and MCH, which include but are not limited to dynorphin, enkephalin, cocaine and amphetamine regulated transcript (CART), nesfatin-1, corticotropin releasing factor (CRF), neurotensin, and galanin (Skofitsch and Jacobowitz, 1985; Fallon and Leslie, 1986; Broberger, 1999; Watts et al., 1999; Goebel et al., 2009; Hahn, 2010). Retrograde tracing with pseudorabies virus showed that roughly one-third of AcbSh-projecting pfLH and lLH neurons contain orexin and one-third contain MCH (Kampe et al., 2009), leaving the remaining third uncharacterized. Other anatomical work corroborates these pfLH/lLH to AcbSh connections; moderate amounts of receptors for orexin and MCH are located in the AcbSh as are fibers containing these peptides (Bittencourt et al., 1992; Trivedi et al., 1998; Saito et al., 2001; Baldo et al., 2003). In contrast, the nearby vlLH has far fewer of these peptidergic neuron types, though it contains neurons possessing either vesicular GABA transporter or, to a lesser degree, vesicular glutamate transporter 2 (Vong et al., 2011). In summary, the pfLH and lLH can potently modulate both aAcbSh and pAcbSh activity using peptide neurotransmitters whereas the vlLH can directly regulate pAcbSh activity, but not aAcbSh activity, using glutamate and GABA.

Some functional evidence reveals how the LH may influence food intake and food-directed effort via direct innervation of the AcbSh. Injection of orexin A into the AcbSh will induce feeding and locomotion (Thorpe and Kotz, 2005). Also, as intra-accumbal orexin administration increases phasic dopamine efflux into the AcbSh (Patyal et al., 2012), orexin likely influences movement patterns and potentially motivated behaviors. In contrast, MCH knockout mice display sensitized dopamine responses via the AcbSh and increased locomotion (Pissios et al., 2008), implying that MCH normally inhibits certain movement patterns. Interestingly, these mice display increased responding to non-rewarding stimuli in a conditioned reinforcement paradigm (Sherwood et al., 2012), which indicates that MCH inhibits actions that are not tied to the receipt of rewards such as food. Sherwood et al. (2012) noted that this process may occur via the AcbSh due to MCH receptor associations with AcbSh dopamine receptors and control over AcbSh neuron excitability (Georgescu et al., 2005; Sears et al., 2010), both of which modulate appetitive responses. Considering that these two peptides are secreted by the LH to the AcbSh, and considering the behavioral effects of orexin and MCH in the AcbSh, the LH is poised to differentially regulate food intake and motivated behaviors via the AcbSh.

Numerous other neuropeptides exist in the LH, and some of these neurotransmitters may also directly regulate AcbSh activity and thus food intake. CART frequently co-localizes with MCH in LH neurons (Broberger, 1999), so CART can be co-transmitted with MCH to the AcbSh. AcbSh injection of CART decreases both regular and AcbSh muscimol-elicited food intake, and CART mRNA levels are decreased within the medial pfLH during fasting (Yang et al., 2005), suggesting that the pfLH may secrete CART to the AcbSh to suppress feeding. Also, recent evidence shows dynorphin injection into the AcbSh alters the hedonic value of sucrose solution (Castro and Berridge, 2014a), and dynorphin can be co-transmitted to the AcbSh by LH orexin neurons (Chou et al., 2001). These other peptide neurotransmitters secreted by the LH are poised to regulate feeding and food-seeking behavior via the AcbSh.

AcbSh input of one other neurotransmitter, CRF, has been associated with modulation of food-directed behaviors. Medial pAcbSh CRF microinjections increase cue-triggered operant responding for sucrose, indicating that AcbSh CRF increases incentive salience of cues that predict availability of palatable food (Peciña et al., 2006). This behavioral effect of AcbSh CRF action is likely mediated by increased AcbSh dopamine release (Lemos et al., 2012). However, prior severe stress prevents this CRF-mediated dopamine release and decreases appetitive behaviors (Lemos et al., 2012). Further, intra-AcbSh CRF administration also decreases sucrose solution intake in two-bottle tests and increases AcbSh acetylcholine (Chen et al., 2012b). Increased AcbSh acetylcholine has been implicated as a marker of satiety (Mark et al., 1992) and may regulate the avoidance of food or decreased motivation to feed (Hoebel et al., 2007). Also worth noting is that AcbSh CRF injection induces oral stereotypy in the absence of foods or objects to interact with (Holahan et al., 1997). This mechanism implicates AcbSh CRF neurotransmission in orofacial motor pattern control. It is unclear whether these AcbSh CRF effects are mediated specifically by LH CRF input, though such input may have feeding suppressive effects. LH CRF neurons are activated by dehydration, and are involved with dehydration-induced anorexia (Watts et al., 1999; de Gortari et al., 2009).

Lastly, the LH transmits glutamate and GABA to the AcbSh. Orexin neurons utilize glutamate as a co-transmitter as they possess vesicular glutamate transporters (Rosin et al., 2003), whereas MCH neurons express glutamic acid decarboxylase, implicating GABA as their co-transmitter (Elias et al., 2008). LH LepRb neurons could also secrete GABA to the AcbSh (Patterson et al., 2011). Through pfLH and lLH innervation of both the aAcbSh and pAcbSh (Kampe et al., 2009), glutamate via orexin neurons and GABA via MCH neurons can modulate the effort to consume foods and the hedonic value of foods. In contrast, the vlLH usually secretes glutamate and GABA without orexin or MCH as co-transmitters. Immunohistochemical and *in situ* hybridization evidence shows vesicular GABA transporter expression in this region (Vong et al., 2011). Glutamatergic and GABAergic projections arising from the vlLH and terminating in the pAcbSh are poised to directly regulate avoidance and aversive qualities of food (Reynolds and Berridge, 2001, 2002; Faure et al., 2010). Ultimately, each route by which LH subregions alter activity of the AcbSh and change food intake is subsequently re-directed back through the LH, as ablation or inhibition of the LH halts AcbSh-mediated feeding (Stratford and Wirtshafter, 2012b; Urstadt et al., 2013a).

## Indirect LH to Acb pathways

Considering the food-directed motivational and hedonic effects of GABA, glutamate, and peptide neurotransmission within the AcbSh, it is of much interest to determine the extra-accumbal sources of such input. Additionally, identifying the afferents to rostral vs. caudal AcbSh regions reveals how other brain regions differentially regulate the motivation to eat and the rewarding or aversive value of foods. Notable inputs to the AcbSh include glutamatergic efferents from the prefrontal cortex, paraventricular thalamic nucleus (PVT), hippocampus, and amygdala (Walaas and Fonnum, 1980; Christie et al., 1985, 1987; Moga et al., 1995) and subcortical GABAergic efferents from the lateral septum (LS), VP, and ventral tegmental area (Churchill and Kalivas, 1994; Van Bockstaele and Pickel, 1995; Zhao et al., 2013). Various neuropeptides are co-transmitted in these inputs. Importantly, the LH innervates all of these brain regions (Goto et al., 2005; Hahn and Swanson, 2010).

There are many brain regions that indirectly connect the LH and the AcbSh. However, this review focuses on select subcortical regions that can “relay” signals from the LH to the AcbSh. As much work has already focused on how the VTA is one relay in LH to AcbSh signaling (Sharf et al., 2010), we instead discuss similar intermediary roles for the LS, VP, and PVT. These regions were selected to address their understudied roles in AcbSh-mediated food intake regulation and to highlight recent research that has begun to suggest these roles. These areas possess orexin and MCH fibers and show expression of receptors for one or both of these neurotransmitters (Bittencourt et al., 1992; Marcus et al., 2001; Saito et al., 2001; Fadel and Deutch, 2002; Baldo et al., 2003), indicating that they receive LH innervation. Additionally, tract tracing evidence reveals direct projections from the LH to these three regions (Chen and Su, 1990; Cullinan and Záborszky, 1991; Risold and Swanson, 1997b; Goto et al., 2005; Kirouac et al., 2006; Hahn and Swanson, 2010). Each of these areas projects to the AcbSh. The nuances of these projection patterns, as well as the functional evidence for their roles in relaying information from the LH to the AcbSh to shape feeding behavior, are discussed below.

An important point to note is that in this review we term these intermediate brain regions in the LH to AcbSh circuit as “relays”. This term gives an overly simplistic impression of their purpose in food intake regulation. Instead of merely relaying signals between LH and AcbSh, they integrate and process signals both from the LH and from their various other afferents. For instance, some authors directly state that the LS had long been referred to as a relay node in affective signaling, whereas it performs far more complex and integrative tasks (Sheehan et al., 2004). For the sake of brevity we refer to these areas as relays, but these regions integrate, process, and transmit other information, such as stress, emotional state, and sensory input, to various other brain areas. Each of these processes can impact food intake.

### The lateral septum

Similar to the nearby accumbens, the LS contains medium-sized spiny neurons. Interestingly, most LS neurons are projection neurons that also colateralize on other LS neurons, allowing them to regulate intra- and extra-LS signaling (Sheehan et al., 2004). LS neurons are primarily GABAergic (Zhao et al., 2013). The LS is intricately subdivided based on neurotransmitter input; intra-LS compartments are distinguished by intensity of fibers labeled for somatostatin, neurotensin, enkephalins, dopamine beta-hydroxylase, tyrosine hydroxylase, calcitonin gene-related peptide, CRF, and other neurotransmitter markers (Risold and Swanson, 1997a). In particular, the caudal LS, distinguished by heavy somatostatin fiber immunoreactivity, is bidirectionally connected with the LH, whereas the rostral LS and ventral LS are bidirectionally connected with medial and anterior hypothalamic sites, respectively (Risold and Swanson, 1997b).

The LS is typically associated with fear and defensive reactions, as manipulations of the LS modify behaviors to fearful stimuli. One well-described phenomenon is LS lesion-induced “septal rage”, an exaggerated defensive behavioral state (for review, see Sheehan et al., 2004). However, the LS also impacts food intake in multiple ways. First, the LS interacts with the viscera. Gastric distention excites or inhibits LS neurons, the appetitive gut hormone ghrelin excites LS neurons, and intra-LS ghrelin increases gastric clearance, allowing for consumption of more food (Gong et al., 2014). Second, the LS regulates neophagia, an avoidance of unfamiliar foods. LS histamine infusion reduces neophagia (Chee and Menard, 2013), as do LS lesions (Chee and Menard, 2011). Third, the LS controls food-directed motivation. LS electrical stimulation increases operant responding to obtain food (Altman and Wishart, 1971), while large LS lesions cause sporadic meal bout interruptions due to hyperactivity and diversion of efforts to grooming and exploration (Flynn et al., 1986). More recent work reveals that the ventral LS may be the locus for food-directed behavior. For instance, ventral LS lesions curb neophagia while dorsal LS lesions do not (Chee and Menard, 2011). Also, the inclusion of sucrose into a restricted feeding schedule diet induces c-fos expression in the ventral LS more robustly than in the dorsal LS (Mitra et al., 2011). These data implicate the LS in various aspects of feeding.

**Figure 2:**
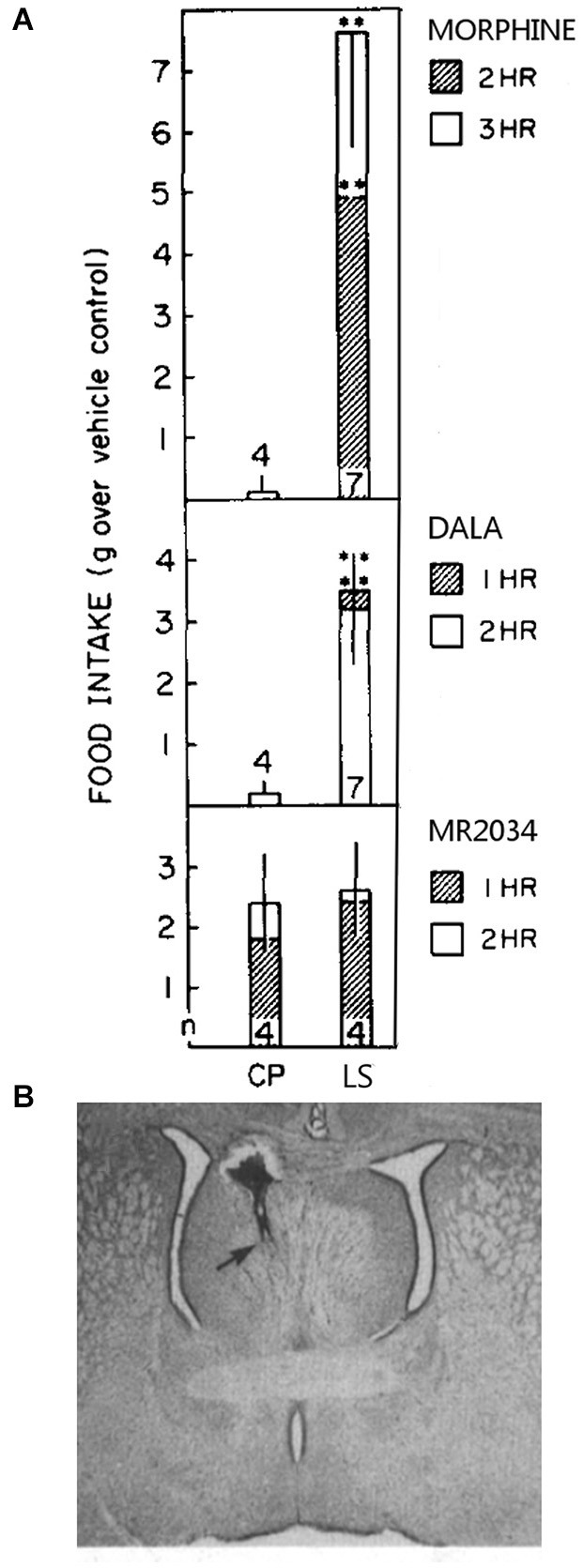
**Morphine and DALA increase food intake significantly above controls when injected into the caudal LS but not into the nearby caudate putamen (CP) (panel A)**. MR-2034 did not significantly increase intake in either of these brain areas. Numbers at the base of each bar represent the number of animals used per group. A Nissl-stained section with an injection site into the the border of the caudal LS and septofimbrial area is shown in panel **(B)**. These figure components were adapted from a prior study (Stanley et al., 1988).

Particularly important is that the LS regulates food intake via opioid receptors. Injection of morphine or the delta receptor agonist (D-Ala^2^)-Met-enkephalinamide (DALA) into the caudal LS increases in food intake in satiated rats, but LS administration of the broad spectrum opioid receptor agonist MR-2034 (Johnson and Pasternak, 1983) does not affect intake, as shown in Figure 2 (Stanley et al., 1988). Also, fasting promptly decreases leucine-enkephalin (Leu-Enk) fiber density in the LS (Kovács et al., 2005), where Leu-Enk is the endogenous ligand for mu and delta receptors. The authors propose that the decrease in apparent fiber density may be from increased release of Leu-Enk into the LS, implying that enkephalin input to the LS is a feeding signal. As fasting also increases NPY and galanin fiber density in the LS (Kovács et al., 2005, 2007), NPY and galanin input may shape this process. The LH is one potential source of LS enkephalin, galanin, and NPY input (Chronwall et al., 1985; Fallon and Leslie, 1986; Laque et al., 2013).

As mentioned before, the LS and the LH innervate each other (Deller et al., 1994; Risold and Swanson, 1997b; Duva et al., 2005). Fluorogold deposits in most LS subregions label neurons in the LH, and LH Fluorogold infusions label many LS neurons. Further, both orexin and MCH fibers innervate the LS, and the LS expresses their receptors (Bittencourt et al., 1992; Marcus et al., 2001; Saito et al., 2001; Baldo et al., 2003). CART fibers also innervate the LS (Janzsó et al., 2010); some of these fibers likely originate from LH neurons co-expressing MCH (Broberger, 1999) or potentially from LepRb neurons co-transmitting GABA (Patterson et al., 2011; Laque et al., 2013). It is unclear whether orexin, MCH, CART, or GABA neurotransmission to the LS can directly affect food intake, though orexin can control food anticipatory behavior. Orexin neurons in the pfLH and lLH exhibit increased c-fos expression in animals anticipating a meal during a restricted feeding paradigm (Jiménez et al., 2013), and orexin expression in the LH is upregulated when this meal is a sweetened chow diet (Olszewski et al., 2009). Similarly, meal anticipation in food-restricted rats increases c-fos expression in the ventral LS, especially those given access to sucrose with their chow (Mitra et al., 2011). Thus, pfLH/lLH orexin neurons, operating potentially via the ventral LS, influence expectations of palatable meals when food availability is limited.

LS regions receiving LH innervation project to the AcbSh. The caudal LS, which is the primary LS recipient of LH input (Risold and Swanson, 1997b), sends terminals throughout the AcbSh with some LS neurons innervating the aAcbSh and many more innervating the pAcbSh (Brog et al., 1993). Particularly noteworthy is that the rostral LS projects to the aAcbSh (Zahm et al., 2013). As LS neurons are GABAergic (Zhao et al., 2013) and co-express either dynorphin or enkephalin (Fallon and Leslie, 1986; Harlan et al., 1987), caudal LS input to the whole AchSh is poised to retune food-directed behaviors, and rostral LS input specifically to the aAcbSh could potentiate hedonic valuation of tastes and food consumption (Reynolds and Berridge, 2002; Faure et al., 2010; Castro and Berridge, 2014a). Further, the LS can generally inform the AcbSh of predicted availability of regular and palatable meals. By interacting with the LS, the LH can control various aspects of AcbSh-mediated feeding.

### The ventral pallidum

The VP, a basal forebrain region, has recently become an area of much interest in the research fields of drug abuse and food intake. This region shares some of the properties of its more rostral neighbor, the accumbens, in that it also possesses medium spiny neurons (Kupchik and Kalivas, 2013). However, more caudal and lateral areas of the VP have medium-sized *aspiny* GABAergic neurons that fire spontaneously. Further, although the VP has cholinergic neurons like those in the accumbens, VP cholinergic neurons project to other brain areas instead of serving solely as interneurons (Carlsen et al., 1985). The entirety of the VP is anatomically identified by a dense Substance P-immunoreactive fiber network, and is subdivided by strong neurotensin and strong calbindin staining in the anteromedial and posterolateral parts of the VP, respectively (Zahm, 1989; Zahm et al., 1996). As the VP diagonally traverses the basal forebrain from rostromedial to caudolateral (Paxinos and Watson, 2013), we henceforth refer to VP subdivisions as anteromedial (amVP) or posterolateral (plVP).

The VP is implicated in the “liking” of foods and the motivation to feed (for detailed review, see Castro and Berridge, 2014b). VP neurons are excited by sucrose reward and cues that predict it (Tindell et al., 2004). VP neurons also increase firing when sucrose solutions are infused into the mouth (Tindell et al., 2006). Interestingly, salt solutions that are aversive to sodium-replete rats do not increase VP neuron firing, but such solutions robustly increase VP neuron firing in sodium-depleted rats (Tindell et al., 2006). This effect highlights the VP’s role in the hedonic evaluation of tastes and how it can be altered by homeostatic signals. Lesions of the VP, and not surrounding structures such as the LH, produce aversion to otherwise pleasurable tastants (Cromwell and Berridge, 1993); such effects are mediated primarily through the plVP (Ho and Berridge, 2014). VP lesions also disrupt the acquisition of food-associated conditioned place preference (McAlonan et al., 1993), indicating a role for the VP in learning about food-associated cues.

Numerous neuropharmacological studies have described behavioral roles of specific neurotransmitter receptors within the VP. VP GABA_A_ receptor activation decreases intake and increases negative orofacial responses to palatable, neutral, and unpalatable solutions (Shimura et al., 2006). Interestingly, this inhibition of the plVP decreases effort to procure palatable food, yet increases chow intake in rats given the choice between sucrose and chow (Farrar et al., 2008). Conversely, VP GABA_A_ receptor blockade increases food intake (Stratford et al., 1999) and causes a robust preference for foods high in fat (Covelo et al., 2014). VP orexin receptor or MOR activation increases “liking” reactions to sweet foods (Smith and Berridge, 2005; Ho and Berridge, 2013). The fact that VP MOR activation increases “liking” reactions may seem paradoxical, given that opioids inhibit VP neurons (Mitrovic and Napier, 1995). However, opioid input can disinhibit VP neurons via inhibition of inhibitory presynaptic inputs (Napier et al., 1992; Kupchik et al., 2014). Collectively, these manipulations indicate that certain neurotransmitters act within the VP to regulate the motivation to procure and the liking of foods, as well as altering macronutrient selection.

Anatomical work links the LH, VP, and AcbSh. LH projections innervate the VP and surrounding basal forebrain areas (Cullinan and Záborszky, 1991; Goto et al., 2005; Hahn and Swanson, 2010). These projections arise from the pfLH and lLH, particularly from orexin neurons (Baldo et al., 2003) and potentially from LepRb neurons (Patterson et al., 2011). Some projections also originate from the vlLH (Cullinan and Záborszky, 1991). The VP in turn projects to the accumbens in a topographic manner (Haber et al., 1985). The amVP projects to the anterior half of AcbSh, the subcommissural VP projects to the AcbC, and the plVP projects to the lateral pAcbSh (Phillipson and Griffiths, 1985; Brog et al., 1993). Most of these VP to AcbSh projections utilize GABA as their neurotransmitter, as suggested by stains for GAD mRNA (Churchill and Kalivas, 1994). These VP neurons may also use opioid peptides as co-transmitters (Fallon and Leslie, 1986; Harlan et al., 1987).

Functional evidence also links these three brain regions. VP electrical stimulation induces c-fos expression in the accumbens, including the aAcbSh (Panagis et al., 1997). MOR activation in the plVP induces increased c-fos expression in the pAcbSh, while MOR antagonism reduces baseline c-fos expression in both aAcbSh and pAcbSh. Further, MOR antagonism in the aAcbSh blunts plVP MOR-mediated food intake and plVP MOR-elicited increases in “liking” of foods (Smith and Berridge, 2007), indicating a VP to AcbSh opioid projection. Paradoxically, though these hedonic “hotspots” functionally interact, they do not directly innervate each other; the amVP “coldspot” innervates the aAcbSh “hotspot” and the plVP “hotspot” innervates the lateral pAcbSh (Phillipson and Griffiths, 1985; Brog et al., 1993). Further investigation of intra-accumbal and intra-pallidal processing may resolve this issue. Additionally, as VP disinhibition induces a preference for fatty foods, it would be interesting to see if VP to AcbSh signaling is involved with fatty food preferences induced by accumbens MOR activation (Zhang et al., 1998).

**Figure 3:**
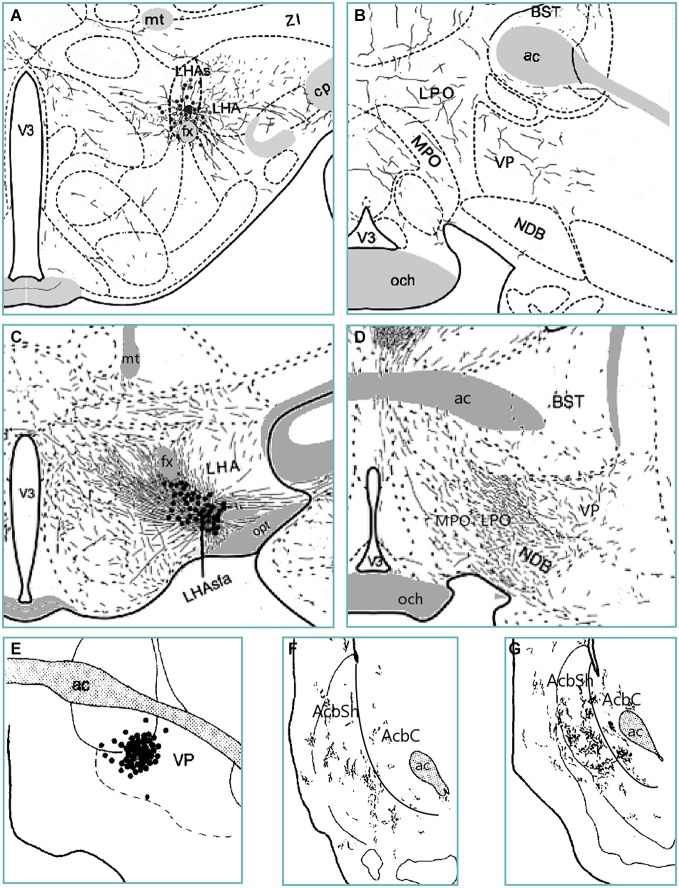
**Anterograde tract tracing evidence from various studies indicates an ascending trans-pallidal LH to AcbSh circuit in the rat brain**. PHA-L-infiltrated neurons in the suprafornical LH (LHAs; panel **A**) and the anterior subfornical LH (LHAsfa; panel **C**) send moderate amounts of fibers to the amVP (panels **B** and **D**) (Goto et al., 2005; Hahn and Swanson, 2010). PHA-L-labeled neurons in the amVP (panel **E**) send projections to the anterior (panel **F**) and especially posterior (panel **G**) medial AcbSh (Groenewegen et al., 1993). Thus, the VP subregion receiving LH input projects in turn to the medial AcbSh. Abbreviations: BST—bed nucleus of the stria terminalis; cp—cerebral peduncle; fx—fornix; LHA—lateral hypothalamic area; LPO—lateral preoptic area; MPO—medial preoptic area; mt—mammillothalamic tract; NDB—diagonal band nucleus; och—optic chiasm; opt—optic tract; V3—third ventricle; VP—ventral pallidum; ZI—zona incerta.

Anatomical evidence suggests that the LH secretes orexin, opioids, and GABA (from LepRb neurons) into the VP (Fallon and Leslie, 1986; Baldo et al., 2003; Patterson et al., 2011; Laque et al., 2013). The VP then sends GABAergic and opioidergic input to the AcbSh (Fallon and Leslie, 1986; Harlan et al., 1987; Churchill and Kalivas, 1994). Indeed, accumbens-projecting VP neurons likely receive input from the pfLH and vlLH (Figure 3). How then may this LH—VP—AcbSh circuit influence feeding? Activation of the LH LepRb neuron population or another GABAergic population can inhibit the VP and suppress intake either by causing foods to become “disliked” or by hindering motivation to obtain foods. In particular, if the LH secretes GABA to the amVP, this would in turn release the aAcbSh from VP inhibition and also suppress feeding. Conversely, orexin or enkephalin input from the LH to the plVP could increase hedonic perception of foods, and can further enhance this effect via activation of the aAcbSh (Smith and Berridge, 2007). Thus, the LH sends homeostatic signals to the VP, which alter the hedonic evaluation of foods and the motivation to procure them. This information is then sent to the AcbSh to transform these motivational and hedonic signals into the initiation or cessation of food procurement and consumption.

### The paraventricular thalamic nucleus

In the rat, the PVT is the most dorsomedial region of the midline thalamus (Paxinos and Watson, 2013). This brain region is comprised of small aspiny neurons. Though behavioral studies discriminate between anterior (aPVT) and posterior (pPVT) subregions, cellular morphology does not clearly reflect this division (Kolaj et al., 2014). PVT neurons are predominantly glutamatergic (Hur and Zaborszky, 2005).

Activation studies examining c-fos expression demonstrate how the PVT, particularly its anterior part, is activated by multiple food-associated and metabolic signals. The PVT tracks the time of day; c-fos expression changes within the aPVT and pPVT based on time of day (Novak and Nunez, 1998). Of particular note is that, in restricted feeding schedules, aPVT c-fos expression increases prior to expected meal availability, and these increases correlate with food anticipatory indicators such as increased locomotion and increased blood corticosterone. These data suggest that the aPVT is a time-keeping oscillator and regulates food anticipatory behaviors in situations of intermittent food access (Nakahara et al., 2004). Further, aPVT activity is also modulated by meal palatability in both restricted and non-restricted paradigms. In restricted feeding schedules, addition of a palatable meal increases food anticipation-associated c-fos expression in the aPVT (Mendoza et al., 2005; Mitra et al., 2011). In ad libitum fed rats, aPVT c-fos expression increases in contexts that signal availability of a saccharin solution (Igelstrom et al., 2010), and aPVT orexin-receptor containing neurons increase c-fos expression in contexts associated with chocolate delivery (Choi et al., 2010). The aPVT also receives metabolic signals; systemic insulin administration and insulin-mediated lard intake increase c-fos within the aPVT but not the pPVT (Warne et al., 2007). These studies show how the aPVT is activated by information about meal timing, availability of palatable foods, and peripheral signals of satiety.

Lesion studies further associate the PVT with food anticipation, food intake, and metabolism. Lesions of the aPVT reduce food anticipatory-mediated increases in blood corticosterone and locomotion without affecting food intake (Nakahara et al., 2004) whereas pPVT lesions increase food intake and body weight, and chronic stress causes this weight gain to shift toward subcutaenous white adipose tissue (Bhatnagar and Dallman, 1999). It should be noted that the pPVT regulates habituation to chronic stressors, as pPVT lesions attenuate this habituation (Bhatnagar et al., 2002). Thus, the aPVT governs food anticipatory signaling and the pPVT regulates food intake, body weight, stress-induced changes in metabolism, and likely stress-mediated eating.

Neuropharmacological evidence examining PVT orexin and GABA input also implicates the PVT in food intake regulation. Orexin 1 receptor knockdown in the pPVT decreases high fat diet consumption without affecting progressive ratio breakpoint for high fat foods in either satiated or food-deprived conditions (Choi et al., 2012). Also, orexin A infusion into the pPVT, but not the aPVT, increases sucrose intake (Barson et al., 2014), and pPVT orexin 1 receptor knockdown decreases high fat chow intake (Choi et al., 2012). As orexin excites pPVT neurons (Ishibashi et al., 2005; Heydendael et al., 2011), it is paradoxical then that pPVT muscimol injection also increases food intake (Stratford and Wirtshafter, 2013). Orexin and GABA may act on different pPVT neuron populations to produce similar food intake effects.

The LH innervates the PVT, as confirmed by anterograde (Goto et al., 2005; Hahn and Swanson, 2010) and retrograde (Chen and Su, 1990; Kirouac et al., 2006; Li and Kirouac, 2012) tracing studies. This hypothalamic projection consists of orexin and its co-transmitter glutamate, verified by PVT orexin receptor expression (Marcus et al., 2001) and orexin fibers within the PVT (Baldo et al., 2003; Parsons et al., 2006). CART fibers also innervate the PVT (Kampe et al., 2009), and such innervation originates partly from the pfLH and vlLH (Kirouac et al., 2006). Although MCH colocalizes with CART in some LH neurons (Broberger, 1999) and the PVT possesses MCH receptors (Saito et al., 2001), few MCH-containing fibers terminate in the PVT (Lee et al., 2014), suggesting that LH to PVT CART innervation does not use MCH as a co-transmitter. Instead, LH LepRb neurons (Patterson et al., 2011; Laque et al., 2013) may secrete CART to the PVT. The PVT also expresses receptors for endogenous opioids and CRF, and the PVT receives CRF input from the pfLH (Minami et al., 1993; George et al., 1994; Potter et al., 1994; Otake and Nakamura, 1995; Ding et al., 1996). Interestingly, LH orexin and CART inputs innervate AcbSh-projecting PVT neurons throughout the PVT’s rostrocaudal axis (Parsons et al., 2006). The concept that the PVT receives hypothalamic CART and orexin signals and sends this information onward to the AcbSh has been proposed previously (Kelley et al., 2005b; Martin-Fardon and Boutrel, 2012). There is also a vlLH projection to the PVT (Chen and Su, 1990) that uses GABA and possibly glutamate (Vong et al., 2011).

In turn, the PVT projects to the AcbSh (Brog et al., 1993; Moga et al., 1995; Parsons et al., 2006; Li and Kirouac, 2008; Vertes and Hoover, 2008; Hsu and Price, 2009). Though initially some evidence indicated that glutamatergic PVT to AcbSh projections innervate cholinergic interneurons (Meredith and Wouterlood, 1990), more recent evidence showed that these PVT to AcbSh projections instead innervate AcbSh medium spiny neurons (Ligorio et al., 2009), though these inputs may still act on cholinergic interneurons via extrasynaptic mechanisms. Both the aPVT and pPVT project to the aAcbSh and pAcbSh (Li and Kirouac, 2008), allowing both PVT regions to influence feeding behavior through both AcbSh subregions. Considering that the PVT secretes glutamate to the AcbSh (Ligorio et al., 2009), inhibition of the PVT presumably halts this glutamatergic input. AcbSh AMPA receptor blockade increases the motivation to eat (Maldonado-Irizarry et al., 1995; Reynolds and Berridge, 2003); this manipulation mimics cessation of AcbSh glutamate input. Further, in non-stressful environments, decreased glutamate release to both the aAcbSh and most of the pAcbSh can induce appetitive behaviors (Reynolds and Berridge, 2008). Thus, PVT inhibition would suppress glutamate release to the AcbSh, allowing for the initiation of food intake.

Although it is likely that multiple glutamatergic inputs, not solely the PVT, regulate AcbSh-mediated feeding, the PVT likely has at least a modulatory role on food intake. Electrophysiological data indicates that although PVT input to the AcbSh produces EPSPs, it does not produce action potentials as other glutamatergic inputs do (O’Donnell and Grace, 1995). However, it remains unclear whether a specific afferent source, or a collection of sources, controls AcbSh glutamate-mediated feeding. Synaptic glutamate levels in the AcbSh decrease during the onset of feeding (Rada et al., 1997), indicating that multiple glutamatergic AcbSh afferents must suppress their input to allow for feeding to occur. Also, inhibition of prefrontal cortical inputs alone does not increase food intake; suppression of additional glutamate inputs is required to initiate feeding (Richard and Berridge, 2013). The PVT may be an additional input whose activity must also decrease in order to decrease AcbSh synaptic glutamate and allow for the initiation of food intake.

The concept of an LH to PVT to AcbSh circuit is supported by the effects of GABA in the PVT and glutamate effects in the AcbSh. Activating pPVT GABA_A_ receptors induces eating (Stratford and Wirtshafter, 2013). Inhibitory input from the LH to the pPVT, via pfLH/lLH CART neurons and their GABA co-transmission, from pfLH opioid-secreting neurons (Brunton and Charpak, 1998), from LH CRF neurons (Siggins et al., 1985), or from vlLH GABA neurons, would halt pPVT excitatory input to the aAcbSh and pAcbSh. By this circuit, inhibitory LH inputs to the pPVT decrease glutamate release in the AcbSh, allowing for feeding to occur.

However, excitation of pPVT neurons, via orexin receptor activation, also induces intake (Barson et al., 2014; Kolaj et al., 2014). How might increased PVT glutamate output to the AcbSh increase food-directed behavior? One explanation may lie in different PVT neuron populations—those mediating GABA-elicited feeding and those mediating orexin-elicited feeding—innervating different postsynaptic targets in the AcbSh. More specifically, PVT neurons regulating GABA-elicited feeding may project to AcbSh projection neurons, whereas PVT neurons regulating orexin-elicited feeding may project to AcbSh GABAergic interneurons. Such differences in PVT neuron projection patterns need to be verified, however.

An alternate explanation is the existence of a presynaptic “brake” on glutamatergic inputs to the AcbSh. Most AcbSh glutamatergic afferents, including the PVT, express the inhibitory mGluR2 presynaptically (Conn and Pin, 1997; Gu et al., 2008), and activation of mGluR2 within the AcbSh decreases glutamate release, an effect that persists for several minutes due to formation of presynaptic long term depression (Kahn et al., 2001; Xi et al., 2002). Robust PVT glutamate release within the AcbSh would then activate PVT mGluR2 autoreceptors or mGluR2 heteroreceptors (on other glutamatergic terminals), suppressing further glutamate release into the AcbSh. PVT-mediated excess glutamate release into the AcbSh that would trip this “brake” can occur from: (1) strong excitation of the pPVT by orexin and glutamate input from the LH (Kolaj et al., 2014), (2) excitation of the pPVT by activation of the aPVT from palatable food contexts and/or metabolic signals (Vertes and Hoover, 2008); and (3) excitation of the prefrontal cortex by PVT neurons that collateralize to both the AcbSh and the prefrontal cortex (Bubser and Deutch, 1998; Otake and Nakamura, 1998). It should be noted that AcbSh neurons have two resting membrane potentials—a slightly depolarized “up” state and a hyperpolarized “down” state (O’Donnell and Grace, 1995; O’Donnell et al., 1999). Although excess glutamate input would elicit action potentials in AcbSh neurons in their “up” state (Lape and Dani, 2004), simultaneous GABAergic inputs from other sources would maintain AcbSh neurons in their “down” state. In situations where GABAergic inputs to the AcbSh drive neurons to their “down” state, excess extracellular glutamate may not induce action potentials and instead may inhibit further presynaptic glutamate release. As a result, inhibitory inputs to AcbSh neurons maintain these neurons in their “down” state and permit the initiation of feeding unhindered by glutamate input. This “presynaptic brake” hypothesis requires substantial verification, however. In particular, it is unclear whether robust PVT glutamate release into the AcbSh shuts down AcbSh glutamate inputs in this manner, and it must be determined whether such a mode of input deactivation specifically induces food-oriented behaviors.

In summary, LH input informs PVT subregions of bodily nutrient states and palatable food availability. Then, this input modulates aPVT-mediated palatable food anticipation and metabolic signaling as well as modifying the pPVT’s motivational signals to procure foods. Finally, these PVT divisions can collectively potentiate food-directed behaviors through the aAcbSh and pAcbSh, particularly in non-stressful familiar environments. Further anatomical study can clarify whether specific PVT neuron subsets, such as those governing GABA-mediated food intake vs. others governing orexin-mediated intake, selectively innervate AcbSh projection neurons or AcbSh interneurons.

## Conclusions

Here we have described multiple ways that the LH can regulate food intake via the AcbSh both directly and indirectly, and can do so by altering various aspects of food-seeking behavior. Multiple subdivisions of the LH differentially innervate the AcbSh and utilize different combinations of neurotransmitters. Neurotransmitters localized in the LH are implicated in food intake and effort to procure foods, and administration of LH-originating neuropeptides or their antagonists into the AcbSh impact such behaviors. Also, the LH projects to numerous other brain regions, some of which project to the AcbSh, regulate its activity, and subsequently influence food-directed behaviors. A collection of the pathways we have described can be found in Table 1. We highlight a subcortical network that serves to regulate aspects of food procurement and consumption.

**Table 1:** **Anatomical evidence for direct projections and their neurotransmitters**.

Projection	Origin of evidence	Neurotransmitters used	Origin of evidence
pfLH → AcbSh, lLH → AcbSh	Phillipson and Griffiths (1985), Brog et al. (1993), Kampe et al. (2009), Hahn and Swanson (2010)	Orexin, MCH*	Bittencourt et al. (1992), Trivedi et al. (1998), Saito et al. (2001), Baldo et al. (2003), Kampe et al. (2009)
vlLH → pAcbSh	Phillipson and Griffiths (1985), Brog et al. (1993), Goto et al. (2005)	GABA	Vong et al. (2011)
pfLH → LS, lLH → LS	Risold and Swanson (1997b)	Orexin, MCH, CART*	Bittencourt et al. (1992), Marcus et al. (2001), Saito et al. (2001), Baldo et al. (2003), Janzsó et al. (2010)
vlLH → LS	Risold and Swanson (1997b)	GABA	Vong et al. (2011)
LS → AcbSh	Brog et al. (1993), Zahm et al. (2013)	GABA	Zhao et al. (2013)
pfLH → PVT, lLH → PVT	Chen and Su (1990), Kirouac et al. (2006), Hahn and Swanson (2010)	Orexin, MCH, CART*	Marcus et al. (2001), Saito et al. (2001), Baldo et al. (2003), Kirouac et al. (2006)
pfLH → PVT	Otake and Nakamura (1995)	CRF	Otake and Nakamura (1995)
vlLH → PVT	Chen and Su (1990)	GABA	Vong et al. (2011)
PVT → AcbSh	Meredith and Wouterlood (1990), Brog et al. (1993), Parsons et al. (2006), Vertes and Hoover (2008), Ligorio et al. (2009)	Glutamate	Meredith and Wouterlood (1990), Ligorio et al. (2009)
pfLH → VP, lLH → VP	Cullinan and Záborszky (1991), Hahn and Swanson (2010)	Orexin*	Baldo et al. (2003)
vlLH → VP	Goto et al. (2005)	GABA	Vong et al. (2011)
VP → AcbSh	Haber et al. (1985), Phillipson and Griffiths (1985), Brog et al. (1993)	GABA	Churchill and Kalivas (1994)

*Certain co-transmitters are assumed for specific neuron types. Specifically, orexin is co-transmitted with glutamate and dynorphin, MCH with GABA, and CART with GABA.

The AcbSh and the LH have a direct bidirectional anatomical connection (Usuda et al., 1998; Kampe et al., 2009). This bidirectional circuit serves to increase feeding in both directions of its signaling. For example, inhibition of the AcbSh disinhibits the LH (Stratford, 2005), in particular orexin neurons (Baldo et al., 2004), and can drive food intake through this descending pathway. On the other hand, the feeding induced by excitation of the LH via glutamate receptor agonists (Stanley et al., 1993a) may act in part by activating orexin, MCH, or other LH neurons (Obukuro et al., 2010; Li et al., 2011) and by increasing their peptidergic output to the AcbSh. Indeed, intra-AcbSh orexin induces feeding while orexin antagonists suppress feeding (Thorpe and Kotz, 2005). Alternatively, anorectic peptides secreted from the LH to the AcbSh such as CART may antagonize the descending AcbSh—LH pathway, as may LH GABA neurotransmission to the pAcbSh. Also, the anterior vlLH receives input specifically from the aAcbSh hedonic hotspot (Thompson and Swanson, 2010), the vlLH region shows increased c-fos expression after aAcbSh inhibition (Baldo et al., 2004), and the vlLH projects back to the pAcbSh with GABA (Vong et al., 2011). This LH feedback may serve to re-tune the hedonic perception of foods. Energy state signals modulate LH activity and reduce feeding by blunting appetite-inducing signals transmitted to the AcbSh, which then inform the LH to avoid or cease intake.

**Figure 4:**
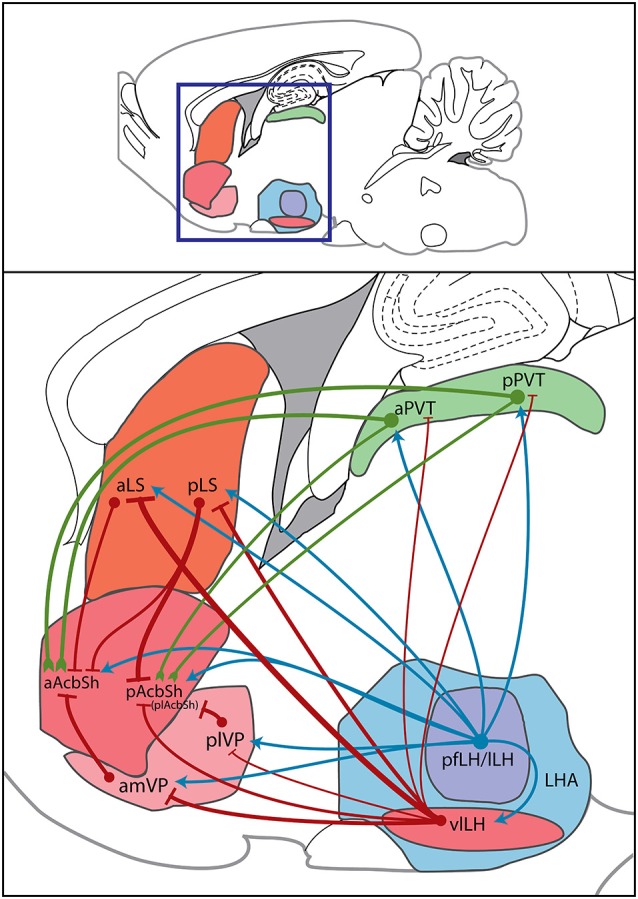
**Sagittal diagram of direct hypothalamic and indirect trans-pallidal, trans-thalamic, and trans-septal innervation of the AcbSh**. Within a sagittal plane, the boxed region designates an area of the rat forebrain within which the regions of interest reside (top panel); this area is magnified to show sources of AcbSh innervation (bottom panel). Subregions of the LH area (LHA), the pfLH, lLH, and vlLH, project both directly to the AcbSh and to other regions that project onward to the AcbSh. Green lines indicate glutamatergic signals, red lines indicate GABAergic signals, and blue lines indicate mixed or neuropeptidergic signals. Circles indicate cell bodies. Line thickness denotes “strength” of connections. Such strengths were determined by amounts of anterogradely labeled fibers or retrogradely labeled cells observed in prior studies of each specific projection. This sagittal template was modified from a brain atlas (Paxinos and Watson, 2013). plAcbSh—posterolateral AcbSh.

Here we have also proposed or added to three potential indirect pathways by which the LH can regulate AcbSh activity, either by modulating the motivation to feed or by changing the reward value of foods. These routes are summarized in Figure 4 and in the following statements.

1. The LH sends projections to the LS (Deller et al., 1994) which contain orexin, MCH, CART, GABA, and potentially other neurotransmitters (Bittencourt et al., 1992; Broberger, 1999; Baldo et al., 2003; Janzsó et al., 2010). Also, opioids that originate from the LH (Fallon and Leslie, 1986) could directly mediate feeding behavior via the caudal LS (Stanley et al., 1988), a region that receives much LH input (Risold and Swanson, 1997b). As such, the LH is arranged to alter LS-mediated food-anticipatory rhythms and food-seeking behavior with homeostatic information (such as food deprivation or sudden availability of palatable food), and can use LS GABAergic and opioidergic outputs to induce feeding via the AcbSh.
2. The plVP receives orexin input from the LH that enhances liking of foods (Ho and Berridge, 2013). VP GABA input and plVP opioid input can directly affect food intake (Smith and Berridge, 2005; Shimura et al., 2006), and such input arises in part from the LH (Fallon and Leslie, 1986; Chou et al., 2001). The VP in turn is poised to inhibit the AcbSh through GABAergic or opioidergic input (Harlan et al., 1987; Churchill and Kalivas, 1994) and may drive food intake via this input. Thus, palatable food availability or homeostatic state signals processed by the LH can influence the hedonic valuation of foods, food-directed effort, and fat preference governed by the VP. This LH to VP input subsequently modulates VP GABAergic and opioid input to the AcbSh and re-tunes food-associated affective and motivated behaviors.
3. LH input can excite the aPVT and pPVT through orexin innervation (Ishibashi et al., 2005), or inhibit these areas via CART, enkephalin, GABA, or CRF (Kolaj et al., 2014). Activation of pPVT orexin and GABA_A_ receptors induces food intake (Stratford and Wirtshafter, 2013; Barson et al., 2014), suggesting that LH neuron groups differentially regulate feeding via the pPVT. Both the aPVT and pPVT in turn send glutamatergic projections to the AcbSh, which, when halted, allows for feeding to occur via both the aAcbSh and pAcbSh in non-stressful situations (Reynolds and Berridge, 2008). Inhibitory input from the LH to the pPVT would suppress glutamate input to the AcbSh. Alternatively, orexin neurons that fire to palatable food cues excite aPVT neurons, which then activate pPVT neurons via intra-PVT glutamatergic innervation, and orexin innervation of the pPVT further enhances this excitation. Excess glutamate release from the pPVT to the AcbSh activates presynaptic autoreceptors or heteroreceptors, which then halt AcbSh glutamate input and induce motivation to feed. By this route, the LH alters PVT activity and subsequently changes food anticipation, food procurement, and food selection, and this alteration of PVT activity modulates food intake through changes in PVT glutamatergic input to the AcbSh.

A few issues arise when interpretting the wealth of aforementioned anatomical data. One issue is that many tracing studies do not specify whether LH projections innervate LS/VP/PVT projection neurons or interneurons. Also, inputs to these brain regions could be en passant, allowing the LH to regulate not only these but other regions receiving collaterals. Further, some anatomical studies using light microscopy rely on visualizing varicosities to suggest innervation of an efferent target, though such innervation may not actually occur in the observed brain region. Visualization of these circuits with electron microscopy can resolve these issues, and further investigation with other methods can properly define the purpose of specific projections in these described circuits. Such data will paint a more complete picture of how energy state integrators impact the motivation to feed and the rewarding value of foods.

Considering the various avenues by which homeostatic and sensory inputs can inform AcbSh-mediated motivational and reward-related processing, how then is such processing executed into action? We have mentioned that AcbSh output is routed back through the LH in order to initiate feeding. Indeed, the LH is required for AcbSh-mediated feeding behavior (Stratford and Wirtshafter, 2012b). However, as the AcbSh translates motivation into the initiation of movement patterns, it does so through multiple efferent targets aside from the LH. Such downstream regions include the globus pallidus, VP, and mesencephalic motor regions, all of which regulate aspects of AcbSh-mediated goal-directed actions (Jones and Mogenson, 1980; Brudzynski et al., 1993; Stratford and Wirtshafter, 2012b). It is via these multiple downstream regions that the AcbSh initiates the seeking of goals such as foods, yet the LH is an essential component of this food intake-controlling network in that its ablation (Stratford and Wirtshafter, 2012b) or its inhibition (Urstadt et al., 2013a) halts AcbSh-mediated feeding and not other behaviors.

Drawing conclusions about brain structure functions from lesion and microinjection studies can be difficult if careful scrutiny is not paid to the specific anatomical spaces involved. Overlaying “empirical” functional spaces with native chemoarchitecture and connectivity maps in future studies is absolutely essential for delineating the exact neural substrates by which certain aspects of a behavior, such as food intake, are regulated (Khan, 2013). Towards this goal, we present and add to a subcortical framework formed from functional studies, activation maps, and connectivity patterns showing how the LH interacts directly and indirectly with the AcbSh. Through this arrangement of connections, the LH can directly and indirectly alter food-oriented behaviors governed by the AcbSh. In turn, the AcbSh sends signals to motor effectors and the LH to initiate the vital behavior of feeding.

## Conflict of interest statement

The authors declare that the research was conducted in the absence of any commercial or financial relationships that could be construed as a potential conflict of interest.
